# Quantification Approaches for Fatigue Crack Resistance of Thermoplastic Tape Layered Composites with Multiple Delaminations

**DOI:** 10.3390/ma14061476

**Published:** 2021-03-17

**Authors:** Anastasiia Khudiakova, Andreas J. Brunner, Markus Wolfahrt, Gerald Pinter

**Affiliations:** 1 Polymer Competence Center Leoben GmbH, Roseggerstraße 12, 8700 Leoben, Austria; anastasiia.khudiakova@pccl.at (A.K.); markus.wolfahrt@pccl.at (M.W.); 2 Retired from Empa, Swiss Federal Laboratories for Materials Science and Technology, Laboratory for Mechanical Systems Engineering, Überlandstrasse 129, 8600 Dübendorf, Switzerland; andreas.brunner@empa.ch; 3 Institute of Materials Science and Testing of Polymers, Montanuniversität Leoben, Otto Glöckel-Straße 2/II, 8700 Leoben, Austria

**Keywords:** automated tape placement, interlayer bonding, mode I fracture, double cantilever beam, multiple delaminations, crack branching, damage index

## Abstract

Automated tape placement with in-situ consolidation (ATPisc) is a layer-wise manufacturing process in which the achievement of proper interlayer bonding constitutes one of the most challenging aspects. In the present study, unidirectional carbon fiber reinforced thermoplastic laminates were produced following different manufacturing protocols using ATPisc. The interlayer bonding of the laminates produced was characterized by mode I fatigue fracture tests with double cantilever beam (DCB) specimens. Independent of the manufacturing approach, the laminates exhibited multiple cracking during DCB testing, which could not be evaluated simply following standard methods. Thus, various data analysis methodologies from literature were applied for the quantitative assessment of the fracture behavior of the laminate. The examination of the evolution of the damage parameter φ and the effective flexural modulus throughout testing enabled a better understanding of the damage accumulation. The Hartman-Schijve based approach was revealed to be a convenient method to present fatigue crack growth curves of laminates with multiple delaminations. Moreover, a preliminary attempt was made to employ a ‘zero-fiber bridging’ methodology to eliminate the effect of additional damage processes on the fatigue crack growth that resulted in large-scale, partially massive fiber bridging.

## 1. Introduction

Carbon fiber reinforced polymer (CFRP) laminate composites have been increasingly adopted in global industries, such as aerospace and automotive, due to their light weight leading to fuel savings and lower CO_2_ emissions. They are also known for their high strength-to-weight and stiffness-to-weight ratios, giving them an edge over traditional metal parts [1,2]. Composites with thermoplastic matrices are the most attractive for industry sectors because of their outstanding fracture toughness, high temperature resistance, and recyclability [3,4]. Moreover, thermoplastics exhibit the unique ability to solidify upon cooling from the melting temperature without undergoing chemical crosslinking [5]. Therefore, thermoplastic composites usually do not require post-processing, such as heat or pressure, which leads to lower production costs and significant energy savings. One of the advanced methods to produce CFR thermoplastic laminates is automated tape placement with in-situ consolidation (ATPisc). 

ATPisc is an additive manufacturing method, where a continuous carbon fiber reinforced thermoplastic tape is heated with, e.g., a diode laser up to the melting temperature of the matrix and placed on a mold using a compaction roller. The intimate contact created between the tape and the roller ideally leads to inter-diffusion of the polymer chains across the bond interface followed by material solidification with subsequent cooling [6]. The non-isothermal layer-wise manufacturing of ATPisc makes the interlayer bonding a critical aspect of the laminates. Therefore, many research works have been devoted to the investigation of the influence of manufacturing parameters on the delamination resistance of ATPisc laminates [7,8,9,10,11]. For this purpose, they have applied the mode I double cantilever beam (DCB) test. However, only quasi-static loading has been addressed so far, while the crack resistance of ATPisc laminates under cyclic loading has not yet been studied. Since service loads are typically cyclic rather than quasi-static, the respective fatigue fracture characterization requires intensive research. 

The present work attempts to close this gap by characterizing the fatigue delamination resistance of unidirectional (UD) carbon fiber reinforced polyphenylene sulfide (CF/PPS) laminates produced by ATPisc. The laminates were manufactured following two different manufacturing protocols with an additional application of two manufacturing parameter sets of the tape placement speed and process temperature. Unexpectedly, besides the main mid-plane crack propagation, multiple interlayer delaminations were visually observed for several specimens during fatigue mode I DCB testing. It is known that additional damage processes affect the main mid-plane crack propagation, leading to data that could not be reasonably quantified according to the standard data analysis [12,13,14]. Pascoe et al. [15] have shown that multiple delaminations of different lengths yield different local strain energy release rate (SERR). In addition, it was shown that the position of these delaminations in a specimen effect both the local and global SERR. The problem of multiple delamination in laminates has also been addressed by [16,17,18] numerically. Hence, an important goal of this study was to find ways to quantitatively characterize the laminate quality from the experimental point of view. For this purpose, the evolution of the crack length correction factor Δ and the effective flexural modulus was examined throughout the tests. In addition, the damage parameter φ [19] was calculated to characterize the degree of damage at the beginning and the end of testing. The fatigue delamination resistance was characterized using both, the classic Paris relation and the modified Hartman-Schijve equation [20]. In addition, three-point bending tests were performed on specimens prior to and after DCB testing to determine a possible change in the flexural modulus. Moreover, a ‘zero-fiber bridging’ methodology for the determination of ‘conservative’ mode I delamination resistance data [21] was applied as a preliminary attempt to exclude the effect of multiple cracking and fiber bridging on the fatigue crack propagation.

The paper has the following outline. First, the methods used to produce laminates are described. Then the tests performed, namely fatigue mode I DCB and three-point bending, as well as the data reduction methods applied are presented. Among those were the Paris-relation, the modified Hartman-Schijve equation, the ‘zero-fiber bridging’ methodology, and calculations of damage parameters. The results obtained are presented in the Results and Discussion section. They include visual and microscopic analyses of the laminates before and after testing, fatigue crack growth curves, and the evolution of damage parameters throughout testing. Finally, the Summary, Conclusion, and Outlook section summarizes the work done.

## 2. Materials and Methods

### 2.1. Materials and Laminate Manufacturing

The laminates were produced out of a continuous carbon fiber reinforced polyphenylene sulfide (CF-PPS) tape with a width of 26.4 mm and a thickness of 0.14 mm. The tape was placed on a flat tool using ATPisc, forming unidirectional laminates of 24 layers. 

**Figure 1:**
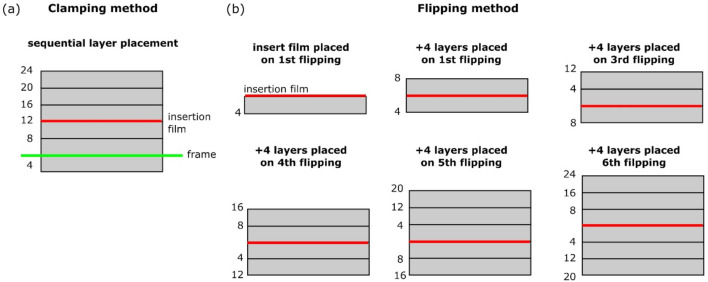
Schematic drawing of (**a**) the clamping and (**b**) the flipping method. The insert film is marked in red, the frame is marked in green. The fiber direction is into the page.

The laminates were produced following two different manufacturing approaches, clamping and flipping (Figure 1). According to the first approach, the first four layers placed on the flat tool were clamped with a picture frame made out of aluminum profiles (Figure 1a). The remaining 20 layers were placed on each other within the frame. According to the second approach, the laminates were flipped after placement of every four layers until 24 layers were placed in total (Figure 1b). A polyimide insert film (from Ube Industries, Yamaguchi, Japan) with a thickness of 12.5 µm was placed in the mid-plane of every laminate to create a pre-crack for DCB testing. Thus, the clamping approach provided a manufacturing process without interruptions, except one for placing the insert film. Whereas, six process interruptions were made during the flipping-manufacturing. In addition, two different manufacturing sets of tape placement speed and process temperature were applied −5 m/min and 330 °C, and 10 m/min and 350 °C. The manufacturing protocols used are summarized in Table 1. With respect to the nomenclature of the tests, the letters ‘c’ and ‘f’ refer to the manufacturing methods, clamping and flipping. The first number after the letter refers to the tape placement speed, and the second number to the process temperature. For example, a panel produced at 10 m/min and at 350 °C following the clamping approach is called c-10-350.

### 2.2. Fatigue Mode I DCB Testing

**Figure 2:**
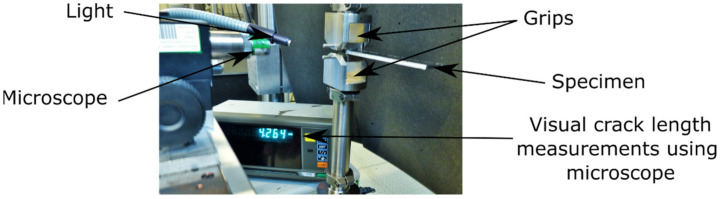
Test setup of the fatigue mode I double cantilever beam (DCB) testing.

Rectangular DCB specimens with dimensions of 200 mm × 20 mm × 3.2 mm were cut out of the laminates using a waterjet cutting machine. Steel load-blocks were glued to all specimens using a two-component adhesive (Scotch-weld DP 490, 3M, Maplewood, MN, USA). Before gluing, the load-blocks and corresponding specimen surfaces were grinded with a sandpaper (grit size of 100) and then cleaned with isopropanol. The specimens with the load-blocks glued to them were placed in an oven for 2 h at 65 °C to cure the adhesive. In order to facilitate the visual detection of delamination onset, the side surface of the specimens was covered with a thin layer of a white correction fluid. Fatigue tests were performed on an electro-dynamic test machine (type Instron E3000, from Instron, Norwood, MA, USA) equipped with a 250 N load cell. The test setup is shown in Figure 2. Three specimens per laminates were tested “as received” under standard laboratory conditions (23 °C air temperature, 50% relative humidity). 

The tests were carried out according to a round robin test protocol developed by the European Structural Integrity Society (ESIS) [22,23,24]. Prior to fatigue loading, the specimens were loaded at 1 mm/min until the pre-crack propagated 1–5 mm beyond the tip of the insert film. The displacement value at which pre-cracking was stopped was taken as the $\delta_{max}\;$ value for fatigue loading. The cyclic tests were performed at a frequency of 5 Hz and an $R_{\delta }$-ratio ($\delta_{min}/\delta_{max}$) of 0.1 under displacement control until either a number of 10^6^ cycles or a crack growth rate of about 10^−6^ mm/cycle was reached. The mid-plane delamination was read during short stops at the mean displacement ($\frac{\delta_{min}+\delta_{max}}{2}$) using a travelling microscope. Maximum and minimum values of load and displacement ($P_{max},\;\delta_{max}$ and $P_{min},\;\delta_{min}$) were recorded by the test machine. The values were recorded every 10 cycles up to 1000, every 100 cycles up to 10,000, every 500 cycles up to 50,000, and every 1000 cycles until the end of the test. Such cycle intervals were used to obtain more data points at the beginning of testing, when the most crack growth occurs under displacement control. 

Additionally, a series of fatigue tests was performed on one DCB specimen of c-10-350 type as a preliminary attempt to check the applicability of the ‘zero-fiber bridging’ methodology proposed for eliminating the effects of large-scale fiber bridging in fatigue mode I tests [13,21] to laminates with multiple cracking. According to this methodology, the specimen was first pre-cracked under quasi-static loading at a rate of 1 mm/min, yielding $\delta_{max}$ for the following fatigue fracture loading with a $R_{\delta }$-ratio of 0.1. Upon reaching a crack growth rate of about 10^−6^ mm/cycle, fatigue fracture loading was terminated. After that, the specimen was quasi-statically loaded again to propagate the crack further, giving a new value of $\delta_{max}$ for the next fatigue loading. In total, this procedure was repeated four times.

### 2.3. Three-Point Bending

Three-point bending testing was performed on pristine specimens and specimens after fatigue mode I DCB testing to examine a possible change in flexural modulus $E_{1}$ prior to and after testing. The tests were performed on a universal testing machine (type Zwick Z010 from Zwick GmbH & Co. KG, Ulm, Germany) equipped with a load cell of 10 kN. The tests were carried out in accordance with ASTM D 790 [25] and under standard laboratory conditions on specimens without any conditioning. The span to thickness ratio of 50:1 was chosen as large as possible for the specimen length of 200 mm. The loading and support noses had radii of 5 mm. The formulae for the loading rates and flexural modulus can be found in the test standard [25]. The pristine specimens refer to original DCB specimens of the same dimensions that were not tested yet. The specimens first tested under fatigue loading were then cryo-fractured along the mid-plane, resulting in two individual beams of the same thickness. These individual beams were used for three-point bending tests.

### 2.4. Optical Analysis

**Figure 3:**
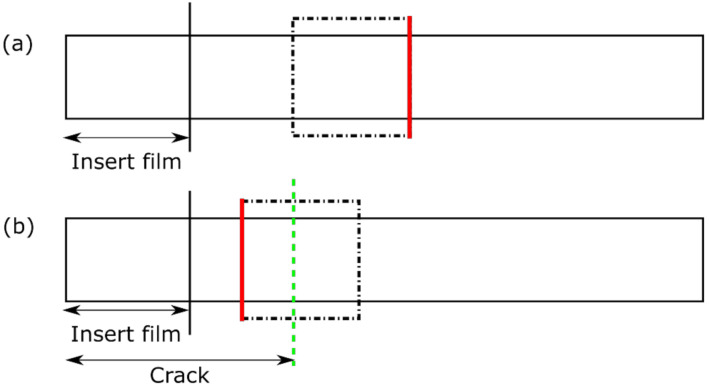
Schematic illustrations of the samples extracted out of the DCB specimens for the microscopic examinations: (**a**) Pristine specimens; (**b**) specimens after fatigue mode I DCB testing. The view is on the top specimen surfaces. The green dashed lines refer to the final crack tip after fatigue testing. The black dot-dashed lines refer to the samples cut out of the DCB specimens, with the cross-section surfaces examined using the optical microscope marked in red. The figure is not scaled.

Optical analysis of polished specimen cross-sections was performed before and after fatigue loading in order to investigate damage. The micrographs were acquired using the optical 3D measurement system (type Alicona InfiniteFocus from Alicona Imaging GmbH, Raaba, Austria). The cutting schematic of the samples is illustrated in Figure 3. 

### 2.5. Data Analysis Techniques 

#### 2.5.1. The Paris Relation and the Modified Hartman-Schijve Equation

The fatigue crack growth curves are presented using the classic Paris relation (Equation (1)) and the modified Hartman-Schijve equation [26] (Equation (2)). The latter was applied to try to collapse the curves into the single ‘master’ curve [27].

(1) $$\frac{da}{dN}=M*{\sqrt{G_{Imax}}}^{s},$$

where a is the crack length, N is the number of cycles, M and s are material parameters $,\;G_{Imax}$ is the strain energy release rate calculated with $P_{max}$ and $\delta_{max}$. M corresponds to the intersection of the linear region of the Paris-plot with Y-axis, and  s is its slope. The crack growth rate $\frac{da}{dN}$ was calculated using the nine-point incremental polynomial technique as described in ASTM E 647 [28].

(2) $$\frac{da}{dN}=D{\left[\frac{\sqrt{G_{Imax}}-\sqrt{G_{Imax,thr}}}{\sqrt{1-\frac{\sqrt{G_{Imax}}}{\sqrt{A}}}}\right]}^{n},$$

where D, n are the constants of the power law, D corresponds to the intersection of the linear fit of the data with Y-axis, and  n describes its slope. The terms $\sqrt{G_{Imax,thr}}$ and A are chosen so that the plot of Equation (2) becomes virtually linear [27]. A has units of J per $m^{2}$ and can be first estimated to be about of the critical quasi-static SERR, which can be further refined in order to achieve the best linear fit [26].

It should be noted that some studies have used ($\Delta \sqrt{G_{I}}=\sqrt{G_{Imax}}-\sqrt{G_{Imin}}$) instead of $\sqrt{G_{Imax}}\;$ in Equation (1) and in the numerator of Equation (2) [20,26,29,30,31,32]. However, in the present work, several specimens have exhibited extremely low $P_{min}$ around 0.35 N, which could not be properly measured by the load cell of 250 N used. In order to be consistent with data reduction for all laminates, only maximum values were used for all calculations in order to avoid a usage of questionable minimum load values. Therefore, $\sqrt{G_{Imax}}$ was used in both Equations (1) and (2). Such a form of equations has also been used in [33,34,35].

#### 2.5.2. Calculations of $G_{Imax}$

$G_{Imax}$ was calculated according to either corrected beam theory (CBT) (Equation (3)) or effective crack length method (ECLM) (Equation (4)).

(3) $$\begin{array}{c} G_{Imax}=\frac{3P_{max}\delta_{max}}{2b\left(a+\left|\Delta \right|\right)}\frac{F_{displ}}{N_{block}}\\ F_{displ}=1-\frac{3}{10}{\left(\frac{\delta }{a}\right)}^{2}-\frac{3}{2}\left(\frac{\delta l_{1}}{a^{2}}\right)\\ N_{block}=1-{\left(\frac{l_{2}}{a}\right)}^{3}-\frac{9}{8}\left[1-{\left(\frac{l_{2}}{a}\right)}^{2}\right]\frac{\delta l_{1}}{a^{2}}-\frac{9}{35}{\left(\frac{\delta }{a}\right)}^{2} \end{array}$$

(4) $$G_{Imax}=\frac{3P_{max}\delta_{max}}{2ba_{eff}}\frac{F_{displ}}{N_{block}}$$

(5) $$a_{eff}=\;\frac{h}{2}{\left(\frac{E_{1}bC}{N_{block}}\right)}^{1/3},$$

where Δ is the crack length correction factor, b is the specimen width, $F_{displ}$ is the large-displacement correction, $N_{block}$ is the load block correction, $l_{1}$ is the distance between the center of loading pin to the mid-plane of the half-beam of the DCB specimen, $l_{2}$ is the distance between the center of the loading pin to the edge of load block, h is the thickness of one beam, and $E_{1}$ is the flexural modulus. For analysis of every fractured specimen, a value of $E_{1}$ was taken to be equivalent to that obtained from a three-point bending test performed on these specimen beams after fatigue testing. 

The crack length correction factor Δ was determined from ${\left(C/N_{block}\right)}^{1/3}$ plotted versus crack length a as the absolute value of the intersection of the linear fit with the negative X-axis (Equation (6)). Δ was set to zero in case of a positive intercept [36]. Three different definitions of a were used, namely (i) $a_{m}$ visually observed with a travelling microscope during testing, (ii) $a_{calc}$ back-calculated from the machine compliance using Equation (7), and (iii) $a_{eff}$ calculated using ECLM (Equation (5)). The compliance calibration enables calculation of the crack length taking into account changing of the specimen compliance C automatically measured by the test machine [37]. While ECLM enables calculations of $a_{eff}$ using C and an independently measured flexural modulus $E_{1}$, and accounts for the fracture process zone and associated crack tip effects [38].

(6) $$\begin{array}{c} {\left(C/N_{block}\right)}^{1/3}=A_{0}+A_{1}a\\ \Delta =-\frac{A_{0}}{A_{1}}, \end{array}$$

where C is the compliance, $N_{block}$ is the load block correction, and $A_{0}$ and $A_{1}$ are parameters of the linear fit.

(7) $$C=D*a^{m},$$

where D is a constant and m is the exponent of the power law.

In addition, the effective flexural modulus $E_{1}$ was calculated at each data point for every specimen using Equation (8).

(8) $$E_{1}=\;\frac{8N_{block}{\left(a+\left|\Delta \right|\right)}^{3}}{Cbh^{3}}$$

#### 2.5.3. Damage Parameter φ

The degree of damage in the specimens was characterized using the damage parameter φ, which corresponds to the reduction factor of transverse and shear stiffness in Equation (9) [19,39]. It has a value in a range between 0 and 1, where ‘0′ implies the total loss of transverse and shear stiffness and ‘1′ the absence of damage. For φ=1, Δ takes on a value of $\Delta_{elastic}$ (Equation (11)). φ was calculated using Equation (10) deduced from Equation (9) for ϑ = 0.3 [19,39].

(9) $$\chi^{2}=\;{\left(\frac{\Delta }{h}\right)}^{2}=\frac{1}{10}\left(\frac{E_{1}}{\varphi G_{12}}-2\vartheta \right)+0.24\sqrt{\frac{E_{1}}{\varphi E_{2}}}$$

(10) $${\left(\frac{\Delta_{elastic}}{h}\right)}^{2}=\frac{1}{10}\left(\frac{E_{1}}{G_{12}}-2\vartheta \right)+0.24\sqrt{\frac{E_{1}}{E_{2}}}$$

(11) $$\varphi ={\left(\frac{0.12}{\chi^{2}+0.06}\sqrt{\frac{E_{1}}{E_{2}}}\left(1+\sqrt{1+7\left(\chi^{2}+0.06\right)\frac{E_{2}}{G_{12}}}\right)\right)}^{2}\;\mathrm{for}\;\vartheta =0.3,$$

where $E_{2}$ is the transverse modulus, $G_{12}$ is the shear modulus, and ϑ is the Poisson’s ratio. The calculations of φ were performed using the flexural modulus $E_{1\_3pb}$ obtained from three-point bending tests. $E_{2}$ and $G_{12}$ were determined using the Reuss model for each laminate type (see the calculations in the  Supplementary Materials for details) [40].

## 3. Results and Discussion

### 3.1. Laminates After Manufacturing

**Figure 4:**
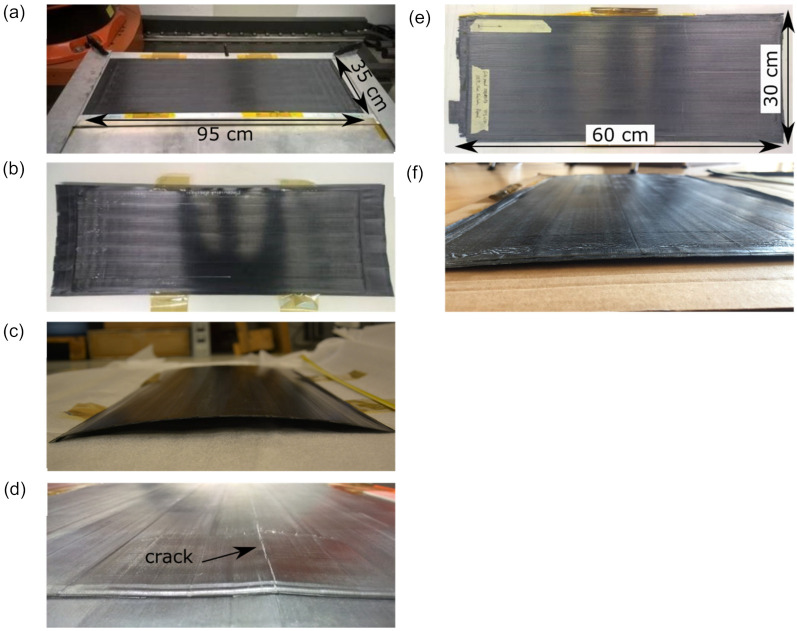
The laminates produced by the clamping method: (**a**) Fixed on the table, (**b**) top, and (**c**) side views after removing the frame, and (**d**) a closer view on the crack due to residual stresses in c-5-330 and the flipping method: (**e**) Top and (**f**) side views.

Figure 4 demonstrates clamping- and flipping-laminates manufactured. The laminates produced by the clamping method exhibited a parabolic curvature after removing the frame. This could be explained by thermal residual stresses, the accumulation of which was caused by temperature gradients throughout the laminate thickness due to layer-wise manufacturing [41,42]. The farther from the uppermost layer, which is subjected to the laser heat, the temperature of the layers is lower [43,44]. This implies that the bottom layers, which have already solidified, constrain the shrinkage of the upper layers during their cooling down. It should be noted that residual stresses have reached such a high level in c-5-330, that transverse cracking of the laminate occurred (Figure 4d). This was not observed for the c-10-350 laminate. The flipping method yielded flatter laminates compared to the clamping method (Figure 4e,f). This could be attributed to a reduced heat build-up within the laminate due to periodic process interruptions made to flip the laminate, so residual stresses did not accumulate or accumulated to a much lesser extent. It is also possible that the residual stresses accumulating during manufacturing within every four layers could be balanced by residual stresses by the following laminate flipping and heat and pressure application. The printing speed and the process temperature did not show an observable impact on the laminate curvature of flipping-laminates.

**Figure 5:**
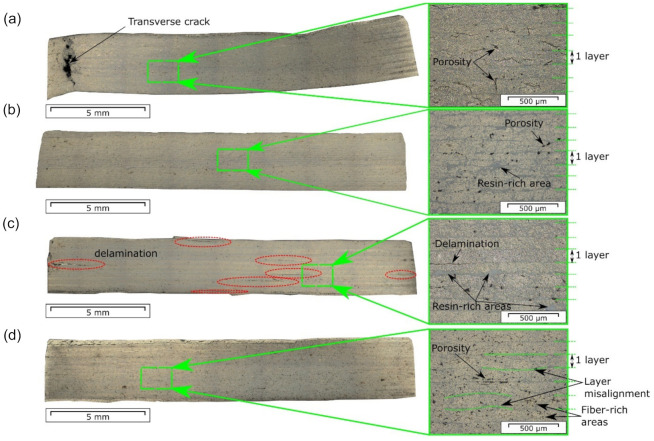
Micrographs of the laminate cross sections made prior to testing for: (**a**) c-5-330-01; (**b**) c-10-350-02; (**c**) f-5-330-04; (**d**) f-10-350-05. The red ellipses point out the interlayer delaminations.

The micrographs of the laminate cross sections made prior to testing are shown in Figure 5. Regions with high porosity concentration forming delaminations within individual layers (Figure 5a) and also between the layers (Figure 5c) were found. Both resin-rich and resin-poor regions were observed in the specimens (Figure 5c,d). Areas with non-uniform fiber distribution and misalignment of the layers were also detected (Figure 5d). The optical analysis also revealed the presence of multiple interlayer delamination in f-5-330, marked in red in Figure 5c. On the one hand, these delaminations could indicate a poor level of interlayer bonding formed during laminate manufacturing. On the other hand, they could also possibly be an artefact from cutting and preparing of the laminate cross-sections, but, nevertheless, still reflecting weak interlayer bonding.

### 3.2. Damage Processes during Fatigue Testing

**Figure 6:**
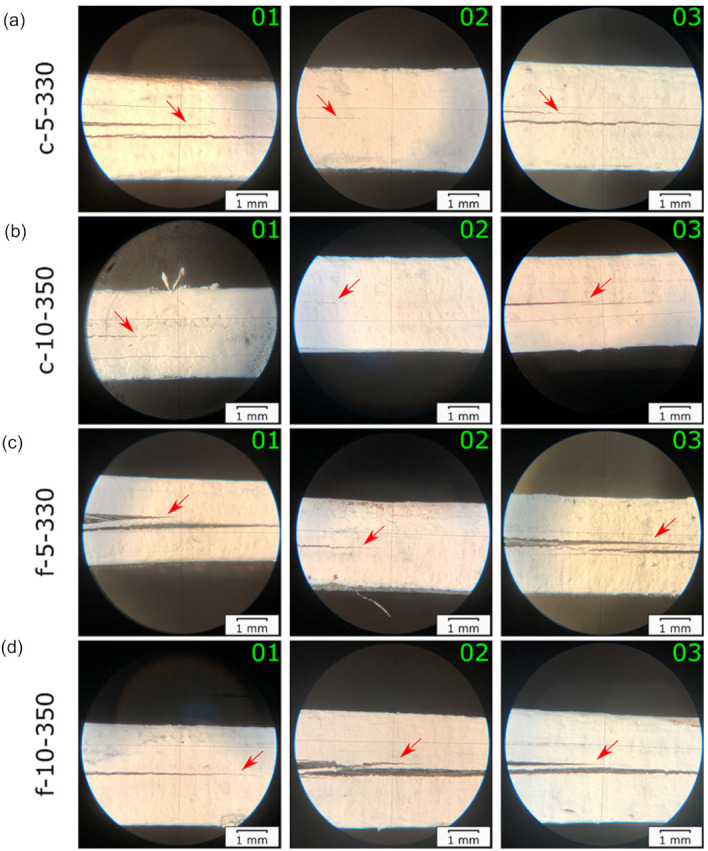
Illustrative images of delamination growth in: (**a**) c-5-330; (**b**) c-10-350; (**c**) f-5-330; (**d**) f-10-350 tested under fatigue. Three specimens for every laminate are presented and sequentially numbered with 01, 02, and 03. The pictures were taken when the crack length reached about 40 mm. The red arrows point at the mid-plane delamination.

**Figure 7:**
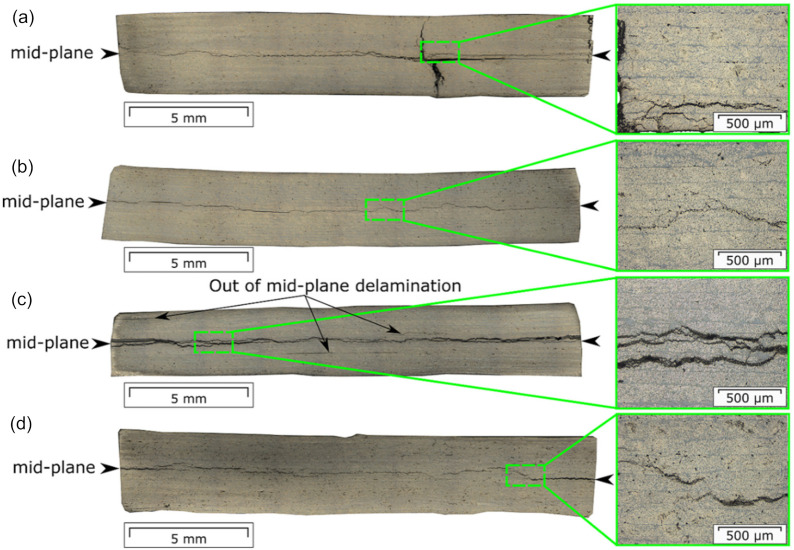
Micrographs of the polished cross sections of selected specimens after fatigue testing: (**a**) c-5-330-01; (**b**) c-10-350-03; (**c**) f-5-330-01; (**d**) f-10-350-02. The black arrows point at the mid-plane delamination.

The delamination behavior visually observed in the specimens during fatigue fracture testing is shown in Figure 6. Two out of three specimens of each laminate type exhibited additional interlayer delamination growth parallel to the mid-plane. Among the specimens displaying a visually observable single mid-plane crack growth were c-5-330-02, c-10-350-02, f-5-330-02, and f-10-350-01. The microscopic analysis, performed on selected specimens, revealed the deviation of the crack from the mid-plane to the adjacent layers (Figure 7). Such a behavior could be caused by porosity entrapped inside the layers [45]. The voids lead to the formation of the intralayer cracks, guiding the crack growth into the other layer and back to the main-plane. Moreover, thermal residual stresses accumulated during laminate manufacturing can be also responsible for the crack deviation [46]. A transverse cracking was found in c-5-330-01, which was a source for additional interlayer cracks (Figure 7a). The multiple interlayer delamination was observed in f-5-330-01, which was already present in not-tested specimens (Figure 5c and Figure 7c). 

### 3.3. Three-Point Bending Tests

The results of three-point bending tests performed on pristine DCB specimens and specimens after fatigue loading are presented in Table 2. The pristine specimens exhibited fairly low deviations in the results obtained for every laminate type, indicating the consistency of the specimens with each other. Although the specimens showed a varying extent of multiple cracking during fatigue tests, there was no significant difference in their $E_{1\_3pb}$. The flexural moduli of the specimens after fatigue testing were also very close to the values of the pristine specimens. All flexural moduli of pristine specimens exhibited a standard deviation within 5% of the respective average. This can be interpreted as reasonable, but not excellent, quality (high-quality CFRP can get down to about 2% scatter/standard deviation in modulus [47]. After fatigue testing, the highest scatter is found for the 350-type specimens (both f and c) rather than the c-5-330 type, whereas f-5-330 tends to be higher (around 9%), but not as much as the 350-type specimens. The absence of a pronounced change in the modulus values determined before and after fatigue testing could indicate that most of the damage affecting the flexural modulus was already induced in the laminates during their manufacturing (Figure 5). On the other hand, these findings could also indicate that the three-point bending modulus is not sufficiently sensitive to delaminations in the beam. Compression in the thickness direction and shear of the layers with respect to each other may “close” some of the delaminations, making $E_{1\_3pb}$ less sensitive to existing damage.

### 3.4. Crack Length Correction Factor Δ and Effective Flexural Modulus $E_{1}$

**Figure 8:**
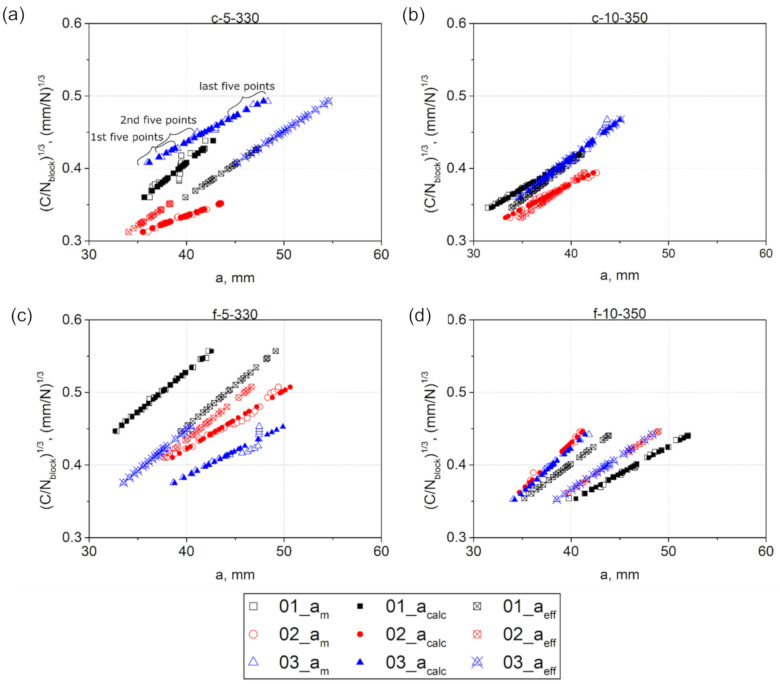
Plots of ${\left(C/N_{block}\right)}^{1/3}$ versus a, where a is either $a_{m}$, $a_{calc}$, or $a_{eff}$ for: (**a**) c-5-330; (**b**) c-10-350; (**c**) f-5-330; and (**d**) f-10-350. The legend refers to every plot.

**Figure 9:**
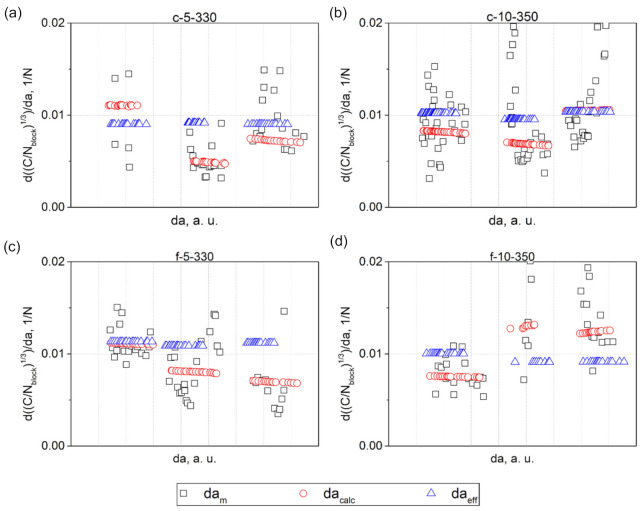
The values of the slopes of linear regressions fitted to datasets of five consecutive points for: (**a**) c-5-330; (**b**) c-10-350; (**c**) f-5-330; and (**d**) f-10-350. 01-03 numbers refer to serial numbers of the specimens. The legend applies to every plot.

Figure 8 shows the comparison of the cube root of the corrected compliance plotted versus (i) $a_{m}$ visually measured during testing, (ii) $a_{calc}$ calculated from the machine compliance using Equation (7), and (iii) $a_{eff}$ calculated using Equation (5). In order to check the linearity of the data obtained, linear regressions were fitted to every dataset out of five consecutive points. The results obtained are presented in Figure 9.

The slope-values of the linear regressions fitted to the visual data exhibited a pronounced scatter, indicating deviation of ${\left(C/N_{block}\right)}^{1/3}$ plotted versus $a_{m}$ from linearity. This can be attributed to the effect of additional damage processes on the main mid-plane crack propagation. On the other hand, the deviation from linearity, or part of that, could also be caused by erroneous crack length measurements made by the machine operator using a travelling microscope [48]. In any case, this means that the length correction factor Δ found using the visual data highly depends on the number of points fitted to the linear regression. Interestingly, when the crack length reached about 39 and 48 mm in c-5-330-01 and f-5-330-03, respectively, the compliance continued to increase with the following cyclic loading without yielding a crack increment (Figure 8a,c). This likely implies that although there was no visual crack increment, the specimen compliance increased due to other damage processes occurring in the specimens. In contrast to the visual data, the linear regression slopes of the machine data, obtained for $a_{calc}$, gradually decreased with the crack growth, or stayed nearly constant (Figure 9). Whereas the data, obtained for $a_{eff}$, had a constant slope over the entire range of the crack length for every specimen, due to the linear relationship between ${\left(C/N_{block}\right)}^{1/3}$ and $a_{eff}$ as follows from Equation (5).

In order to estimate the change in the effective flexural modulus $E_{1}$ throughout the tests, Δ was determined at the beginning and end of testing using the machine data obtained for $a_{calc}$. To this end, linear regressions were fitted to the data range of the first and last 2.5 mm of the crack increment, and also to the entire data range. The results of $E_{1}$ obtained are summarized in Table 3. The results of Δ can be found in Table S3 in the Supplementary Information. For specimens with Δ < 0, the absolute value of Δ was higher at the end than at the beginning of testing, indicating that the effective flexural modulus was increasing with the crack growth. Their values of $\Delta_{all\;points}$ were in a range between corresponding $\Delta_{start}$ and $\Delta_{end}$. For the specimens with positive Δ, the effective flexural modulus had a constant value throughout testing since for its calculation a zero value of Δ was used (Equation (8)). The effective flexural moduli of specimens with Δ < 0 significantly exceeded their $E_{1\_3pb}$ obtained using the three-point bending test (Table 2). High absolute values of the crack length correction factor Δ lead to a rapid increase in the values of the effective flexural modulus $E_{1}\;$ because the latter is a cubic function of the crack length (Equation (8)).

### 3.5. Damage Parameter φ

The degree of damage was estimated using the damage parameter φ that was calculated using $\Delta_{start}$, $\Delta_{end}$, and $\Delta_{all\;points}$ obtained in the previous paragraph (Equation (11)). The results of φ are summarized in Table 4. The results of $\chi^{2}\;$ are presented in Table S4 in the Supplementary Information. In order for φ to be in a range between 0 and 1, $\left|\Delta \right|$ should be greater than $\left|\Delta_{elastic}\right|$ estimated to be about 3.7 mm (Equation (10)). Additionally, it was derived from Equation (9) that $\chi^{2}$ smaller than 5.23 yields unphysical values of φ higher than 1. This corresponds to steep slopes of ${\left(C/N_{block}\right)}^{1/3}$ plotted versus crack length a, where the intercept of the linear regression is at a positive crack length value.

For specimens with φ in a range between 0 and 1, $\varphi_{start}$ was slightly greater than $\;\varphi_{end}$, indicating the absence of a progressive damage accumulation in them. It is also interesting to note that the specimens that exhibited a single mid-plane delamination visually observed on the side specimen surface, namely c-5-330-02, c-10-350-02, f-5-330-02, and f-10-350-01, had damage parameters φ smaller than those obtained for the specimens with multiple cracking (Figure 6). This finding highlight that the mid-plane delamination was not a sole damage process occurring in specimens during fatigue loading. With regard to the results of the microscopic analysis of the selected specimens’ cross-sections after fatigue testing, only one of them showed φ in a range between 0 and 1, namely f-5-330-01 (Figure 7d, Table 4).

### 3.6. Fatigue Crack Resistance Curves: The Paris Relation Based Approach

**Figure 10:**
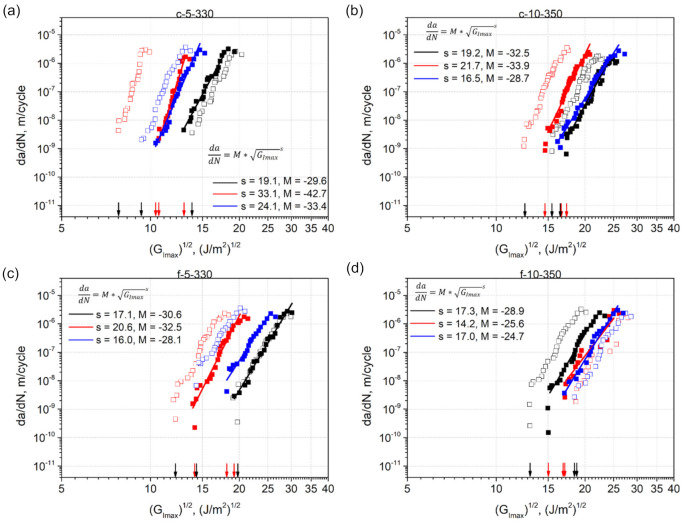
Paris-type plots of da/dN plotted versus $\sqrt{G_{Imax}}$ calculated using CBT with $a_{calc}$ (Equation (3)) and ECLM with $a_{eff\;}$ (Equation (4)) for: (**a**) c-5-330; (**b**) c-10-350; (**c**) f-5-330; and (**d**) f-10-350. The black and red arrows along the X-axis refer to $\sqrt{G_{Imax\_th}}$ of the data calculated using $a_{calc}$ and $a_{eff\;}$, respectively. Black, red, and blue lines refer to linear regressions fitted to the effective data.

In the first step of the fatigue data evaluation, the Paris relation (Equation (1)) was used to present the fatigue delamination resistance using the classic Paris-type plots, where da/dN was plotted versus $\sqrt{G_{Imax}}$. $G_{Imax}$ was calculated using either CBT with $a_{calc}$ (Equation (3)) or ECLM with $a_{eff}$ (Equation (4)). The Paris-like plots obtained are shown in Figure 10. 

It can be seen that the position of the plots along the X-axis varied from specimen to specimen within every laminate set, leading to variations of threshold values $\sqrt{G_{Imax\_th}}$ (Table 5). The smallest standard deviation among the machine data was shown by c-10-350 specimens, in which the lowest extent of damage was visually observed on their side surfaces during testing compared to the other specimens (Figure 6b). Importantly, the machine data calculated with $a_{calc}$ exhibited a larger scatter than the effective data calculated with $a_{eff}$. More precisely, the highest standard deviation among the machine data reached 30% for c-5-330, while the highest standard deviation among the effective data was 16% for f-5-330. The values of standard deviations are given for $\sqrt{G_{Imax\_th}}$, which means that the standard deviation for $G_{Imax\_th}$ is twice as large. Thus, the effective crack length data with a lower scatter was used for further analysis. 

The scatter in the fatigue fracture data of UD carbon fiber reinforced composites is well-known from literature, and was discussed in detail in [49]. The authors have differentiated between intrinsic and extrinsic scatter. With regard to the present work, the former comes from the process induced material morphology of the laminates including, e.g., voids, interlayer delaminations, fiber misalignment, and other defects observed in the laminate micrographs (Figure 5). The extrinsic scatter could be caused by possible deficiencies in the test set-up, erroneous crack length measurements by the machine operator, and variations in the specimen geometries. The scatter of fatigue fracture data for laminates with multiple delaminatons has also been pointed out by Pascoe et al. [15]. In numerous works, e.g., Mujtaba et al. [29], Jones et al. [33], Yao et al. [27], the authors have shown that a fatigue data set with a large scatter can be collapsed into a single linear ‘master’ curve, using the modified Hartman-Schijve equation [20] to present the fatigue data. Therefore, it was decided to apply the Hartman-Schijve based approach in the present research, too.

### 3.7. Fatigue Crack Resistance Curves: The Hartman-Schijve (H-S) Based Approach

The modified H-S equation includes two parameters A and $\sqrt{G_{Imax,thr}}$ that should be defined for every fatigue data set. According to Jones et al. [20], these parameters are (cite) “perhaps best viewed as parameters that are used to ensure that the entire range of data fits the equation”. Further, Jones et al. [26] write that da/dN should be plotted versus $\left[\left(\sqrt{G_{Imax}}-\sqrt{G_{Imax,thr}}\right)/\sqrt{1-\frac{\sqrt{G_{Imax}}}{\sqrt{A}}}\right]$ on a logarithmic scale, taking parameter A (cite) “to be the quasi-static value of the fracture energy, $G_{c}$, or any reasonable first estimate”. Jones et al. [33] further noted that  A corresponds to the quasi-static initiation value of the crack growth, $G_{c0}$. Thereby, A equivalent to $G_{c0}$ was used in the H-S based approach, for example, in [26,27,32]. However, Yao et al. [27] have reported A-values that significantly exceeded the corresponding $G_{c0}$. In the present study, two different approaches were applied to use the modified H-S equation to present the fatigue crack growth curves. 

**Figure 11:**
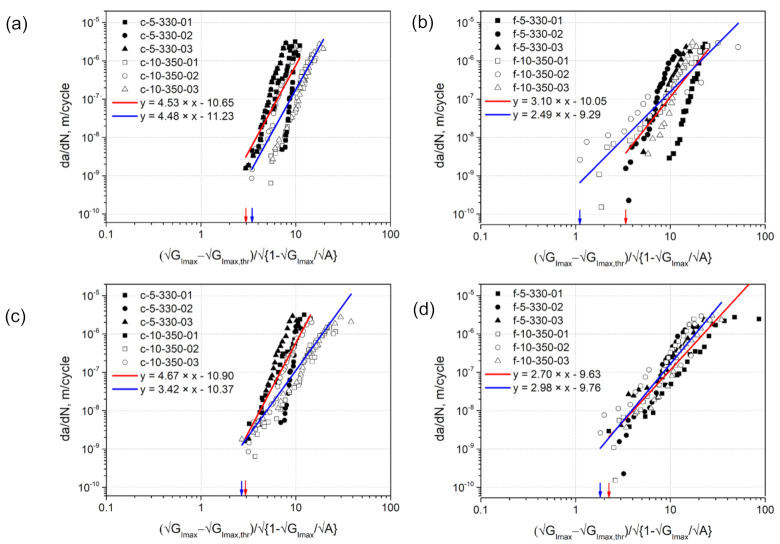
Fatigue crack growth resistance curves obtained using the modified Hartman-Schijve equation, where the parameter A was either varied (**a**,**b**) or kept constant (**c,d**) for every specimen of clamping- (**a**,**c**) and flipping- (**b,d**) laminates. Red and blue lines refer to linear regressions fitted to the data of laminates produced with 5 m/min and 330 °C and to those produced with 10 m/min and 350 °C, respectively.

According to the first approach, both parameters A and $\sqrt{G_{Imax,thr}}$ were varied for every individual test data set in a way that a linear regression fitted to this data reaches the highest R^2^-correlation value. In the second approach, A was kept constant and $\sqrt{G_{Imax,thr}}$ was varied to achieve the highest value of R^2^-correlation of the linear fit. The appropriate parameters were configured automatically by means of a script written in Python. From the formula (Equation (2)), $\sqrt{G_{Imax,thr}}$ must be smaller than the smallest value of $\sqrt{G_{Imax}}$ from a dataset, so that the right-hand member of the equation is positive, while $\sqrt{A}$ must be greater than this value, so that the radicand of the square root is positive. Thus, in the first approach, A was continuously increased from a value a bit higher than $G_{Imax}$ with an increment step of 50 $J/m^{2}$. In the second approach, a value of 950 $J/m^{2}$ was used for A, which was reported in [11] as a reference value for UD CF/PPS laminates produced by ATPisc. A could not be taken to be equivalent to actual $G_{c0}$ values obtained from the quasi-static pre-cracking, because they were smaller than the smallest value of $\sqrt{G_{Imax}}$ for nearly all specimens (Table 5). For every A, $\sqrt{G_{Imax,thr}}$ was continuously increased from a value of 1 $\sqrt{\;J}/m$ with an increment step of 0.05 $\sqrt{\;J}/m$ till the highest value of $\sqrt{G_{Imax}}$. Following this procedure, the highest R^2^- correlation of the linear fits were determined. The results obtained are summarized in Table 6. Figure 11 illustrates the crack growth rate da/dN plotted versus the right-hand component of the modified H-S equation with A and $\sqrt{G_{Imax,thr}}$ from Table 6.

Following the first approach, the highest values of R^2^-correlation of linear regressions were achieved with A-values significantly higher than $G_{c0}$ (Table 6), which can no longer have a physical meaning. Only A of about 690 $J/m^{2}\;$ obtained for specimens f-10-350-01 and -02 were in a comparable range with $G_{c0}$. Remarkably, the second approach where A was kept constant as 950 $J/m^{2}$, which seems to be a reasonable value for the initiation value of the crack growth in UD CF/PPS laminates, yielded the results of $\sqrt{G_{Imax,thr}}$ close to those from the first approach. At the same time, the difference in their values of R^2^- correlation was observed only in the third digit after the decimal point. In addition, the second approach yielded a better agreement of the fatigue crack growth curves of flipping-laminates compared to the first approach (Figure 11). There was no such significant difference observed between the curves of clamping-laminates. These findings highlight the second approach using a constant value of A of 950 $J/m^{2}$ to make overall sense in the application to the laminates investigated.

### 3.8. Zero-Fiber Bridging Approach

**Figure 12:**
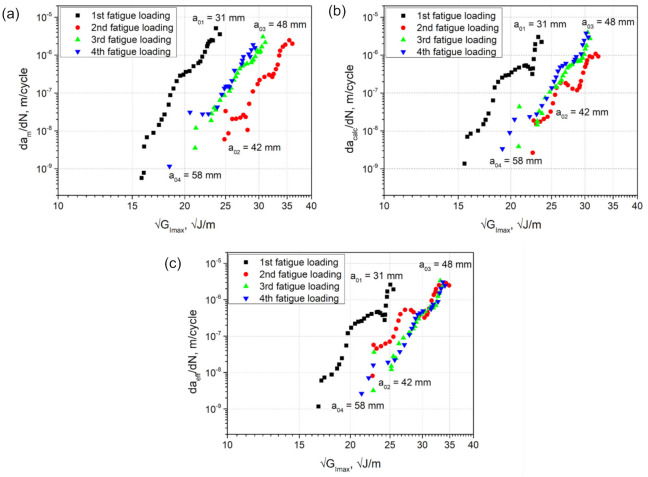
Fatigue crack growth curves of a sequential series of fatigue loadings performed on one DCB specimen (c-10-350) for: (**a**) $a_{m}$ measured visually with the microscope; (**b**) $a_{calc}$ calculated using Equation (2); (**c**) $a_{eff}$ calculated using Equation (3).

The crack growth curves of fatigue fracture loadings sequentially performed on a single DCB specimen of c-10-350 type are shown in Figure 12. In this figure, da/dN is plotted versus $\sqrt{G_{Imax}}$, where a refers to either $a_{m}$ measured visually with the microscope during testing (Figure 12a), $a_{calc}$ calculated using Equation (7) (Figure 12b), or $a_{eff}$ calculated using Equation (5) (Figure 12c). 

According to the ‘zero-fiber bridging’ methodology [21], the fatigue delamination curves shift to the right along the X-axis of the H-S plot when sequential, quasi-static pre-cracking is performed after a given number of fatigue cycles, yielding one mode I H-S-curve length and developing fiber bridging between the specimen beams. The moment when these curves no longer shift to the right, but overlap, corresponds to the fully developed or at least stationary fiber bridging state, i.e., fiber bridging saturation or equilibrium between creation and failure of bridging fibers during delamination propagation.

In the data determined from visually measured delamination lengths, the shift to the right was observed for the second fatigue loading with respect to the first fatigue loading (Figure 12a). This is in agreement with the ‘zero-fiber bridging’ methodology. However, the curve of the third fatigue loading appeared to the left of the second curve. The fourth curve of the last fatigue loading performed nearly overlapped with the third curve, indicating an apparent saturation state. Whether this is really the case requires further quasi-static pre-cracking followed by cyclic fatigue fracture steps. The unexpected shift to the left of the 3rd curve could possibly be caused by a failure of a larger fiber bundle or a more massive “laminate ply bridge” connecting the two specimen beams. After such a breakage, the crack would propagate faster due to a lesser retarding effect of fiber bridging, which would lead to a reduction of $G_{Imax}$. Thereby, two competing mechanisms can be distinguished, i.e., a consistent increase of the typical fiber bridging until a saturation or steady state is reached versus the discrete formation and stochastic failure of “large” fiber or laminate ply bridges. In addition, interlayer delaminations developing during testing influence the bending moments of the specimen beams, which in turn affects the specimen stiffness and the strain energy release rate. Therefore, the “zero-fiber bridging” methodology may not work in case of a discontinuous development of fiber bridging (or of more massive bridging of the main delamination, e.g., caused by multiple cracks) that results in stochastic bridge-breaks and hence does not continuously evolve into a steady-state. 

Interestingly, the shift to the left of the 3rd and 4th curves was not so pronounced for the data of $a_{calc}$ (Figure 12b). Whereas for the effective data of $a_{eff}$, the 2nd, 3rd, and 4th curves overlapped at their upper parts and even exhibited a shift to the right comparing their middle parts (Figure 12c). These findings indicate that the mid-plane crack propagation was significantly influenced by multiple cracking. On the other hand, they could also suggest that, at least to some extent, the visual measurement of the crack length yielded incorrect values during testing. The crack length measurements performed visually using a travelling microscope proved to be very challenging in the case of such a complex crack propagation behavior combined with the sequential series of quasi-static and fatigue loadings.

**Figure 13:**
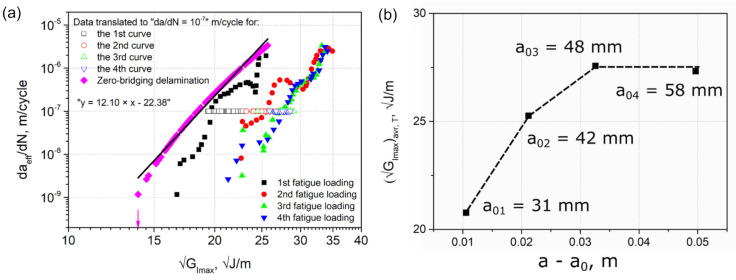
The “zero-fiber bridging” analysis: (**a**) The data translated to an arbitrary value of da/dN = 10^−7^ m/cycle for every curve (unfilled symbols) and the final zero-bridging curve (pink rhombs); (**b**) the average values of the translated data versus the crack length.

The next steps of the “zero-fiber bridging” methodology were performed on the fatigue crack growth curves with $a_{eff}$ (Figure 12c), since they showed the most similar behavior to that described in this methodology [21]. Firstly, the data points of every curve were translated to an arbitrary value of $da_{eff}/dN$ of 10^−7^ m/cycle using Equation (12) (Figure 13). After that, average values ${\left(\sqrt{G_{Imax}}\right)}_{avr,\;T}$ of the data translated were calculated for every curve.

(12) $$\log_{10}\surd G_{Imax,\;T}=\;\frac{1}{m}\left(\log_{10}\frac{da}{dN_{T}}-\log_{10}\frac{da}{dN}\right)+\log_{10}\surd G_{Imax},$$

where $da/dN_{T}$ = 10^−7^ m/cycle, m is the power index in the Paris equation (Equation (1)).

Figure 13b shows a plot of ${\left(\sqrt{G_{Imax}}\right)}_{avr,\;T}$ versus the corresponding crack length, which is described with a non-linear relationship. Alderliesten et al. [21] note that this relationship can be described with a second order polynomial function of $\left(a-a_{0}\right),$ which reaches a horizontal asymptote at the moment of full development of fiber bridging or a steady state. Further, the regression analysis was performed using Equation (13) as described in the methodology. Finally, the zero-fiber bridging curve ($a-a_{0}$ = 0) was obtained then using Equation (14) and presented in Figure 13a. $\sqrt{G_{Imax,thr}}$ deduced from this curve is about 13.9 $\sqrt{\;J}/m$, which is in good agreement with the Hartman-Schijve results for c-10-350 (Table 6).

(13) $$\log_{10}\sqrt{G_{Imax}}=\;C_{0}+C_{1}\left(a-a_{0}\right)+C_{2}\log_{10}\frac{da}{dN}+C_{3}{\left(a-a_{0}\right)}^{2}+C_{4}{\left(\log_{10}\frac{da}{dN}\right)}^{2},$$

where $C_{i}$ are constants obtained through regression, $i=\left[0,4\right]$.

(14) $$\log_{10}{\left(\sqrt{G_{Imax}}\right)}_{a-a_{0}}=\;C_{0}+C_{2}\log_{10}\frac{da}{dN}+C_{4}{\left(\log_{10}\frac{da}{dN}\right)}^{2}$$

It is important to note that the zero-bridging curve was obtained for a single specimen in the present study. Thus, without data from several specimens this curve does not represent the intrinsic scatter coming from material and manufacturing protocols used. Hence, it can be less conservative than the upper-bound curves obtained from using values of $\sqrt{G_{Imax,thr}}$ minus either two or three standard deviations of the mean data described by Jones et al. [33]. However, the single specimen analysis indicates that the zero-fiber bridging procedure works in principle even for DCB specimens with fiber bridging and multiple delaminations. 

## 4. Summary, Conclusions and Outlook

The present study deals with unidirectional CF/PPS laminates produced by automated tape placement with in-situ consolidation (ATPisc) combining two different laminate build-up procedures (labelled “clamping” and “flipping”) with two tape placement speed/control temperature settings (5 m/min and 330 °C versus 10 m/min and 350 °C, basically “low” and “high”, respectively) resulting in four different laminate types. All laminates exhibited multiple interlayer cracking during mode I quasi-static and fatigue fracture testing.

Since there are no established methods for quantifying mode I fatigue delamination resistance when multiple delaminations initiate and propagate, selected approaches that had proven useful in quantifying multiple delaminations for quasi-static mode I loading [50] were adapted and applied to cyclic fatigue fracture and compared. The cube root of the specimen compliance plotted versus the visually observed delamination length usually yields a roughly straight line, the extrapolation of which provides a delamination length correction for root rotation in the quasi-static tests [36]. This line for the Mode I fatigue fracture tests discussed here, however, was not straight, but curved with clearly different slopes at the beginning and at the end. This finding indicates a decreasing, back-calculated flexural modulus of the DCB half beams during the test points at the presence of additional damage processes occurring outside main mid-plane delamination. Three-point bending tests performed on these half beams looking for changes in flexural modulus turned out to not be sensitive enough. Therefore, in order to quantify the degree of damage in each specimen, a damage parameter φ, developed from the analysis of quasi-static Mode I fracture tests [19] was determined. The comparison of φ at the beginning and at the end of testing enabled a better understanding of the damage development and a quantitative comparison for the tested specimens. The fatigue delamination propagation data was then graphically presented as Paris [23,24] and a modified Hartman-Schijve relation [26]. The modified Hartman-Schijve equation was shown to be suitable for the quantitative representation of fatigue crack growth for laminates with multiple delaminations.

Bridging tractions between multiple delaminations propagating in different ply levels involving large fiber bundles, fully or partly still embedded in the matrix, are beyond the resistance effects from large-scale fiber bridging between the two fracture surfaces of a single interlaminar delamination. Hence, a recently proposed methodology for eliminating large-scale fiber bridging effects in Mode I fatigue fracture tests with single mid-plane delaminations [21] was applied to one specimen with multiple cracking for the first time. The result indicated that this method also has potential to deal with more massive Mode I bridging in CFRP laminates.

Clearly, more data have to be generated and analyzed, before a conclusive recommendation on the applicability and potential limitations of the proposed methodologies can be made. The approach presented here, nevertheless, provides a promising road-map for exploring and developing quantitative mode I fatigue fracture characterization of UD carbon fiber reinforced laminates exhibiting multiple cracking. Future research shall also test more specimens from a range of different laminates with thermoplastics and thermoset matrices in order to obtain scatter bands and upper-bound curves required for safe design limits as outlined by Jones et al. [33].

## Figures and Tables

**Table 1:** Manufacturing parameters of unidirectional carbon fiber reinforced polyphenylene sulfide (UD CF/PPS) panels produced by ATPisc.

Specimen ID	Manufacturing Method	Number of Plies	Placement Speed, m/min	Process Temperature, °C
**c-5-330**	clamping	24	5	330
**c-10-350**	clamping	24	10	350
**f-5-330**	flipping	24	5	330
**f-10-350**	flipping	24	10	350

**Table 2:** The flexural moduli obtained from three-point bending tests performed on pristine DCB specimens and on specimens after fatigue mode I DCB testing. The values presented were calculated as the arithmetic mean of four pristine specimens for c-5-330; of five pristine specimens for c-10-350, f-5-330, and f-10-350; and of two specimens tested under fatigue for every laminate type.

	$E_{1\_3pb},\;\mathrm{GPa}$	
Laminate	Pristine Specimens	After Fatigue Mode I DCB Testing
**c-5-330**	111 ± 6 (6%)	109 ± 4 (3%)
**c-10-350**	96 ± 4 (4%)	109 ± 20 (18%)
**f-5-330**	104 ± 3 (3%)	90 ± 8 (9%)
**f-10-350**	112 ± 8 (7%)	100 ± 18 (18%)

**Table 3:** $E_{1}\;$ calculated at the first and last 2.5 mm of the crack increment ($E_{1\;start}$ and $E_{1\;end}$), and at the entire range of the crack length $a_{calc}\;$ ($E_{1\;all\;points}$) for clamping- and flipping-laminates tested under fatigue mode I loading.

Clamping							
5-330				10-350			
Specimen	$E_{1\;start},\;\mathrm{GPa}$	$E_{1\;end},$ GPa	$E_{1\;all\;points},\;\mathrm{GPa}$	Specimen	$E_{1\;start},\;\mathrm{GPa}$	$E_{1\;end},$ GPa	$E_{1\;all\;points},\;\mathrm{GPa}$
**01**	77 ± 1	77 ± 1	77 ± 1	**01**	179 ± 1	192 ± 1	186 ± 1
02 *	639 ± 2	754 ± 2	697 ± 1	02 *	311 ± 1	353 ± 1	331 ± 1
**03**	197 ± 1	236 ± 1	215 ± 1	**03**	96 ± 1	96 ± 1	95 ± 1
**Flipping**							
**5-330**				**10-350**			
**Specimen**	$E_{1\;start}$, **GPa**	$E_{1\;end}$, **GPa**	$E_{1\;all\;points}$, **GPa**	**Specimen**	$E_{1\;start}$, **GPa**	$E_{1\;end}$, **GPa**	$E_{1\;all\;points}$, **GPa**
**01**	99 ± 1	104 ± 1	101 ± 1	01 *	202 ± 1	214 ± 1	208 ± 1
02 *	222 ± 1	250 ± 1	235 ± 1	**02**	70 ± 3	70 ± 3	70 ± 3
**03**	330 ± 1	377 ± 2	352 ± 1	**03**	75 ± 2	75 ± 2	75 ± 2

* A single mid-plane delamination was observed visually on the specimen surface during testing.

**Table 4:** φ calculated using $\Delta_{start}$, $\Delta_{end}$, and $\Delta_{all\;points}$ for clamping- and flipping-specimens tested under fatigue mode I loading.

Clamping							
5-330				10-350			
Specimen	$\varphi_{start}$	$\varphi_{end}$	$\varphi_{all\;points}$	Specimen	$\varphi_{start}$	$\varphi_{end}$	$\varphi_{all\;points}$
**01**	426.171 **	426.171 **	426.171 **	**01**	0.112	0.088	0.097
02 *	0.018	0.014	0.016	02 *	0.066	0.049	0.056
**03**	0.038	0.026	0.031	**03**	391.190 **	391.190 **	391.190 **
**Flipping**							
**5-330**				**10-350**			
**Specimen**	$\varphi_{start}$	$\varphi_{end}$	$\varphi_{all\;points}$	**Specimen**	$\varphi_{start}$	$\varphi_{end}$	$\varphi_{all\;points}$
01 *	0.175	0.143	0.158	01 *	0.279	0.200	0.233
**02**	0.074	0.051	0.061	**02**	417.974 **	417.974 **	417.974 **
**03**	0.044	0.031	0.037	**03**	432.337 **	432.337 **	432.337 **

* A single mid-plane delamination was observed visually on the specimen surface during testing; ** unphysical values of φ greater than 1.

**Table 5:** Quasi-static initiation value of the crack growth $G_{c0}$ and $\sqrt{G_{Imax\_th}}$ of the Paris-type plots showed in Figure 10.

Laminate	$G_{c0}$ , Jm2	$\sqrt{G_{Imax\_th}}$ , Jm2 $\;\mathrm{Using}\;a_{calc}$	$\sqrt{G_{Imax\_th}}$ , Jm2 $\;\mathrm{Using}\;a_{eff}$
**c-5-330**	80.4 ± 64.8 (81%)	10.3 ± 3.1 (30%)	11.4 ± 1.4 (12%)
**c-10-350**	205.4 ± 40.7 (20%)	14.8 ± 2.1 (14%)	16.1 ± 1.4 (8%)
**f-5-330**	366.9 ± 401.1 (109%)	15.4 ± 3.9 (25%)	17.2 ± 2.7 (16%)
**f-10-350**	195.4 ± 58.6 (30%)	16.7 ± 3.2 (19%)	16.4 ± 1.2 (7%)

**Table 6:** A and $\sqrt{G_{Imax,thr}}$ which yield the highest $R^{2}$ -correlation of the linear fits of the fatigue data using the modified Hartman-Schijve equation where (A was varied// A was kept constant).

Specimen	$A,\;J/m^{2}$	$\sqrt{G_{Imax,thr}},\;\sqrt{\;J}/m$	$R^{2}-\mathrm{Correlation}\;$
**c-5-330-01**	5322//950	9.8//10.5	0.9852//0.9845
**c-5-330-02**	5135//950	4.1//5.1	0.9700//0.9698
**c-5-330-03**	5183//950	7.7//8.0	0.9854//0.9849
**Average ± st.dev.**		7.2 ± 2.9 (40%)//7.9 ± 2.7 (34%)	
**c-10-350-01**	5606//950	12.5//14.8	0.9815//0.9792
**c-10-350-02**	5381//950	11.9//12.4	0.9894//0.9888
**c-10-350-03**	5694//950	11.6//14.3	0.9904//0.9863
**Average ± st.dev.**		12.0 ± 0.5 (4%)//13.8 ± 1.3 (9%)	
**f-5-330-01**	5861//950	10.8//17.9	0.9907//0.9707
**f-5-330-02**	5408//950	10.9//11.8	0.9728//0.9719
**f-5-330-03**	5655//950	13.6//16.2	0.9791//0.9779
**Average ± st.dev.**		11.8 ± 1.6 (14%)//15.3 ± 3.1 (21%)	
**f-10-350-01**	652//950	13.8//13.2	0.9713//0.9709
**f-10-350-02**	731//950	16.4//15.9	0.9683//0.9663
**f-10-350-03**	5677//950	11.9//14.8	0.9825//0.9766
**Average ± st.dev.**		14.1 ± 2.2 (16%)//14.6 ± 1.4 (9%)	

## Data Availability

Data is contained within the article or supplementary material.

## Supplementary Materials

The following are available online at https://www.mdpi.com/1996-1944/14/6/1476/s1, Table S1: The transverse and shear moduli calculated for the laminates, Table S2: Fiber weight and volume fractions of the laminates obtained using TGA, Table S3: ∆ calculated at the first and last 2.5 mm of the crack length ($\Delta_{start}$ and $\Delta_{end}$) and at the entire range of the crack length $a_{calc}$ ($\Delta_{all\;points}$) for clamping- and flipping-specimens tested under fatigue mode I DCB loading, Table S4: $\chi^{2}$ calculated using $\Delta_{start}$, $\Delta_{end}$ and $\Delta_{all\;points}$ for clamping- and flipping-specimens tested under fatigue mode I DCB loading.
