# Supplementary material for: Quantification Approaches for Fatigue Crack Resistance of Thermoplastic Tape Layered Composites with Multiple Delaminations
Source: Materials (Basel). 2021 Mar 17;14(6):1476. doi: 10.3390/ma14061476 (PMC8002822; doi:10.3390/ma14061476)
Supplement: Supplementary file 1 [file materials-14-01476-s001.pdf]

materials

Supplementary Information

Quantification Approaches of Fatigue Crack Resistance of Thermoplastic Tape Layered Composites with Multiple Delaminations

Anastasiia Khudiakova [1], Andreas J. Brunner [2], Markus Wolfahrt [1], Gerald Pinter [3], *

[1] Polymer Competence Center Leoben GmbH, Roseggerstraße 12, 8700 Leoben, Austria; anastasiia.khudiakova@pccl.at (A.K.); markus.wolfahrt@pccl.at (M.W.)
[2] Retired from Empa, Swiss Federal Laboratories for Materials Science and Technology, Laboratory for Mechanical Systems Engineering, Überlandstrasse 129, 8600 Dübendorf, Switzerland; andreas.brunner@empa.ch
[3] Institute of Materials Science and Testing of Polymers, Montanuniversität Leoben, Otto Glöckel-Straße 2/II, 8700 Leoben, Austria;
* Correspondence: gerald.pinter@unileoben.ac.at

Citation: Khudiakova, A.; Brunner, A.J.; Wolfahrt, M.; Pinter, G. Quantification Approaches of Fatigue Crack Resistance of Thermoplastic Tape Layered Composites with Multiple Delaminations. *Materials* **2021**, *14*, 1476. https://doi.org/10.3390/ma14061476

Academic Editor: Andrea Spagnoli

Received: 27 January 2021
Accepted: 2 March 2021
Published: 17 March 2021

Publisher's Note: MDPI stays neutral with regard to jurisdictional claims in published maps and institutional affiliations.

Copyright: © 2021 by the authors. Licensee MDPI, Basel, Switzerland. This article is an open access article distributed under the terms and conditions of the Creative Commons Attribution (CC BY) license (http://creativecommons.org/licenses/by/4.0/).

Calculations of the transverse and shear moduli

The transverse and shear moduli were estimated according to Reuss model using Equation S1 and Equation S2.

$$E_2 = \frac{E_{2f}E_m}{E_m f_v + E_{2f}(1-f_v)} \tag{S1}$$

$$G_{12} = \frac{G_{12f}G_m}{G_m f_v + G_{12f}(1-f_v)}$$
$$G_m = \frac{E_m}{2(1+\vartheta_m)} \tag{S2}$$

The following values of Young's modulus and Poisson's ratio of PPS, transverse, and shear moduli of the carbon fiber were used: E_m = 3.45 GPa [1], ϑ_m = 0.38 [2], E_{2f} = 13 GPa [3], G_{12f} = 11.3 GPa [3].

Table 1. The transverse and shear moduli calculated for the laminates.

Laminate	E_2, GPa	G_{12}, GPa
c-5-330	6.01	2.58
c-10-350	5.69	2.39
f-5-330	5.56	2.31
f-10-350	6.20	2.70

The fiber volume fraction f_v was found according to Equation S3 [4].

$$f_v = \frac{w_f/\rho_f}{\frac{w_f}{\rho_f} + \frac{1 - w_f}{\rho_m}} \tag{S3}$$

where ρ_f, ρ_m are the densities of the fiber and matrix, respectively, and w_f is the fiber weight fraction determined using thermogravimetric analysis (TGA).

TGA was performed using the thermogravimetric analyzer TGA/DSC1 Star System (Mettler Toledo, USA). The CF-PPS samples were cut out of the laminates at three equally spaced positions (start, middle, and end of lamination) and had a mass of about 20 mg. Firstly, the specimens were heated from 30 °C to 600 °C at 5 K/min under nitrogen atmosphere in order to perform pyrolysis of the PPS matrix. After that, the samples were cooled down to 200 °C at −20 K/min under the nitrogen atmosphere, and then heated up to 800 °C at 5 K/min under oxygen atmosphere to fully combust the carbon fibers.

The results of w_f values are summarized in Table S2. The corresponding fiber volume fractions f_v were calculated using Equation 11 with fiber density ρ_f of 1.8 g/cm³ and PPS density ρ_m of 1.35 g/cm³ [5].

Table 2. Fiber weight and volume fractions of the laminates obtained using thermogravimetric analysis (TGA).

Laminate	m, mg	w_f, %	f_v, %
c-5-330	20.8±0.1	64.7±2.0	57.9±0.2
c-10-350	20.0±0.6	60.6±1.7	53.6±1.7
f-5-330	20.3±0.6	58.7±0.9	51.6±1.0
f-10-350	20.0±0.4	67.1±0.8	60.4±0.9

Table 3. Δ calculated at the first and last 2.5 mm of the crack length (Δ_{start} and Δ_{end}) and at the entire range of the crack length a_{calc} ($\Delta_{all\ points}$) for clamping- and flipping-specimens tested under fatigue mode I DCB loading.

**Clamping**							
**5-330**				**10-350**			
**Specimen**	Δ_{start}, **mm**	Δ_{end}, **mm**	$\Delta_{all\ points}$, **mm**	**Specimen**	Δ_{start}, **mm**	Δ_{end}, **mm**	$\Delta_{all\ points}$, **mm**
**01**	2.95 **	3.47 **	3.19 **	**01**	−9.95	−11.17	−10.66
**02 ***	−26.48	−30.24	−28.41	**02***	−14.16	−16.39	−15.26
**03**	−18.47	−22.22	−20.32	**03**	0.34 **	0.87 **	0.57 **
**Flipping**							
**5-330**				**10-350**			
**Specimen**	Δ_{start}, **mm**	Δ_{end}, **mm**	$\Delta_{all\ points}$, **mm**	**Specimen**	Δ_{start}, **mm**	Δ_{end}, **mm**	$\Delta_{all\ points}$, **mm**
**01**	−7.26	−8.00	−7.62	**01 ***	−6.02	−7.05	−6.55
**02 ***	−11.47	−13.74	−12.56	**02**	6.27 **	7.27 **	6.72 **
**03**	−14.01	−16.67	−15.28	**03**	5.37 **	6.25 **	5.82 **

* A single mid-plane delamination was observed visually on the specimen surface during testing of these specimens.

** Positive Δ, further Δ=0 was used for the calculations of E_1.

Table 4. χ^2 calculated using Δ_{start}, Δ_{end}, and $\Delta_{all\,points}$ for clamping- and flipping-specimens tested under fatigue mode I DCB loading.

**Clamping**							
**5-330**				**10-350**			
**Specimen**	χ^2_{start}	χ^2_{end}	$\chi^2_{all\,points}$	**Specimen**	χ^2_{start}	χ^2_{end}	$\chi^2_{all\,points}$
**01**	0 **	0 **	0 **	**01**	39.5	49.8	45.4
**02 ***	244.1	318.291	280.9	**02 ***	83.2	111.5	96.6
**03**	118.4	171.344	143.3	**03**	0 **	0 **	0 **
**Flipping**							
**5-330**				**10-350**			
**Specimen**	χ^2_{start}	χ^2_{end}	$\chi^2_{all\,points}$	**Specimen**	χ^2_{start}	χ^2_{end}	$\chi^2_{all\,points}$
**01**	26.0	31.5	28.6	**01 ***	13.3	18.3	15.8
**02 ***	59.7	85.6	71.5	**02**	0 **	0 **	0 **
**03**	87.2	123.5	103.8	**03**	0 **	0 **	0 **

* A single mid-plane delamination was observed visually on the specimen surface during testing of these specimens.

** Since a zero value was used instead of positive Δ, χ^2 that is $(\Delta/h)^2$ was equivalent to zero.

References

1. https://www.bearingworks.com/uploaded-assets/pdfs/retainers/pps-datasheet (accessed on 6 January 2021).
2. https://www.celanese.com/-/media/Engineered%20Materials/Files/Product%20Technical%20Guides/PPS-050_Fortron_DesignManualTG_AM_0913.pdf (accessed on 6 January 2021).
3. Herráez, M.; Mora, D.; Naya, F.; Lopes, C.S.; González, C.; LLorca, J. Transverse cracking of cross-ply laminates: A computational micromechanics perspective. *Compos. Sci. Technol.* **2015**, *110*, 196–204, doi:10.1016/j.compscitech.2015.02.008.
4. Yee, R.Y.; Stephens, T.S. A TGA technique for determining graphite fiber content in epoxy composites. *Thermochimica Acta* **1996**, *272*, 191–199.
5. Liu, D.; Zhu, Y.; Ding, J.; Lin, X.; Fan, X. Experimental investigation of carbon fiber reinforced poly(phenylene sulfide) composites prepared using a double-belt press. *Compos. B Eng.* **2015**, *77*, 363–370, doi:10.1016/j.compositesb.2015.03.062.
